# New System of Instrument Stabilization in Laryngeal Microsurgery

**DOI:** 10.1590/S1808-86942010000500018

**Published:** 2015-10-22

**Authors:** Silvio José de Vasconcelos, Silvio da Silva Caldas Neto

**Affiliations:** 1 MSc in Surgery - UFPE, Substitute Professor of Otorhinolaryngology - Federal University of Pernambuco (UFPE); 2 Associate Professor of Otorhinolaryngology - UFPE. Universidade Federal de Pernambuco

**Keywords:** stabilization, larynx, microsurgery.

## Abstract

**Abstract:**

Laryngeal microsurgery is a kind of treatment for various laryngeal diseases. Because of the need of long instruments and delicate maneuvers, involuntary movements represent relevant difficulty and may be responsible for unintended post-operative results.

**Aim:**

This study proposes a new stabilization system, flexible and versatile, which can significantly reduce involuntary movements made by surgeons.

**Methods:**

This experimental study compared the amplitude of surgeons' involuntary movements with and without the stabilization system. Ten surgeons performed a total of six movements mimicking movements used in laryngeal microsurgery, two of them without the stabilization system. The maneuvers were repeated with the stabilization system and the wire stretched, and after this, the wire was then expanded 3mm and the maneuvers were performed. The average values of the maximum instrument displacement were compared between the groups.

**Results:**

The maximum displacement was higher during the maneuver with the still micro-scissors without the system, when compared with the stabilization system in three different situations. The average was also higher in the maneuver to open and close the micro scissors without the system and with it.

**Conclusion:**

The proposed stabilization system was effective in reducing surgeon shaking in the different situations tested.

## INTRODUCTION

Laryngeal microsurgery is a means to treat numerous laryngeal disorders. It is based on doing surgical procedures through an auto static tube (laryngoscope), placed between the patient's mouth and larynx; the patient is placed on dorsal decubitus, with neck hyperextension. It has enjoyed great development in the last 20 years, thanks to a better understanding of the etiopathogeny and physiopathology of vocal fold disorders, allowing for an increasingly efficient treatment and prevention of laryngeal cancer, as well as the rehabilitation of patients with voice disorders - especially voice professionals. This treatment modality is very relevant in terms of collective health care.

The more laryngeal microsurgery develops; more sophisticated and delicate are its procedures. And for such, there are numerous micro-instruments which are, in general, 22cm long, handled by the surgeon who uses a surgical microscope of variable magnification in order to have the best visualization and illumination of the operating area.

Because of the need for long instruments and the delicate nature of the structures to be manipulated, as well as the surgical gestures to be executed, involuntary movements (shaking) may represent an important difficulty during the procedure, since minimal hand movements by the surgeon are reflected on broad movements at the tip of the instruments. These unintentional movements can be responsible for undesired post-operative results, both from the functional rehabilitation standpoint as well as that of disease control.

Individuals vary in term of how much they shake in the hands or arms. Surgeons have many tactics they use to minimize this problem. Kleinsasser^1^, for instance, recommends supporting the instrument on the lower border to the laryngoscope's proximal end in order to reduce the distance between the fixed point and the tip of the instrument. This effect can be enhanced by using the surgeon's finger to increase the support; however, this can reduce the field of vision of the operating site. Strong and Vaughan suggest to place the forearms in an armrest in order to reduce limb shaking.^2^ Other ergonomic measures can be taken, such as proper patient positioning, a proper design of the surgeon's chair or of the surgical instruments.^3^, ^4^, ^5^ It is also important to observe other measures, such as to avoid caffeine or physical exertion a little while before the surgery. LASER for laryngeal microsurgeries is also another way to reduce shaking; however, it may increase the risk of excessive tissue damage^6^, ^7^, ^8^. In the future, robotics can represent a progress in laryngeal surgery as it happens in other areas of surgery; but such technology is still far from being applicable in practice, and even less considering its commercial availability.

Despite all the above-mentioned measures, it is still impossible to fully eliminate shaking. There is always the need to further reduce the distance between the support point and the tip of the instrument. With this aim, Armstrong et al., in 200^5^,^9^, devised a very simple and efficient instrument stabilization system by introducing an exchangeable “U”-shaped wire that would fit inside the laryngoscope, which would provide the later with an instrument support horizontal rod, located near the distal end of the laryngoscope, thus being able to drastically reduce involuntary movements. However, this system, despite being very efficient, has some drawbacks as to instrument freedom of movement - limiting its use during surgery. In order to rest the instrument on the horizontal rod, the authors designed a notch on the median line. Thus, in the center of the line, the instrument can be well supported. Nonetheless, outside of this notch the instrument can slide freely, increasingly the likelihood of involuntary movements on the horizontal direction. Another inconvenient issue is that the system was made out of steel; therefore it is stiff, not providing for damping of involuntary movements. Moreover, in order to work with the instrument at different points in the vertical direction, it is necessary to change the system for another one which has the horizontal rod at a different height.

Using the same principles adopted by Armstrong et al., in other words, to reduce the distance between the support point and the instrument's tip, the present study aims at developing a new stabilization system, more flexible and versatile, which would enable to cover a greater variety of surgical gestures, and one which provides more stability to the instrument.

## OBJECTIVES

### General

To develop a new instrument stabilization system for laryngeal microsurgery, using a laryngoscope with a nylon wire.

### Specific

• to compare the amplitude of involuntary movements at the tip of the laryngeal microsurgery instruments with and without the stabilization system, trying to maintain the instrument still on the median line;
• to compare the amplitude of involuntary movements at the tip of the laryngeal microsurgery instrument with and without the stabilization system, trying to maintain the instrument still out of the median line.

## METHODOLOGY

### Study Design

This is an experimental study with paired samples.

### Instruments and Equipment

We used a microscissors angled upwards - Microfrance®, for the tests with and without stabilization.

The laryngoscope used was the medium size from Ferrari®.

The tests were all carried out with the help of a Cemapo® surgical microscope with a 400 mm lens and 12.5 mm eye pieces, with a video and digital photodocumentation system coupled to a DVD recorder and a video-monitor with Super-VHS input jack.

### Stabilization System (Figs. 1, 2, and 3)

We made two lateral 1mm holes on the middle point of a vertical line going through the inferior distal border of the laryngoscope. On the laryngoscope's mid-section of the right-side lateral face we fixed a plate graduated between zero and 10 mm. Through these holes we passed a 0.5mm nylon wire. On the left side, the wire's tip was tied to a pin placed next to the proximal end of the laryngoscope. On the right side, the tip of the wire passed through a small hollow cylinder located contralateral to the locked pin of the left side. As it passes through this cylinder, the wire could be fixed to different positions by fixating screws.

**Figure 1:**
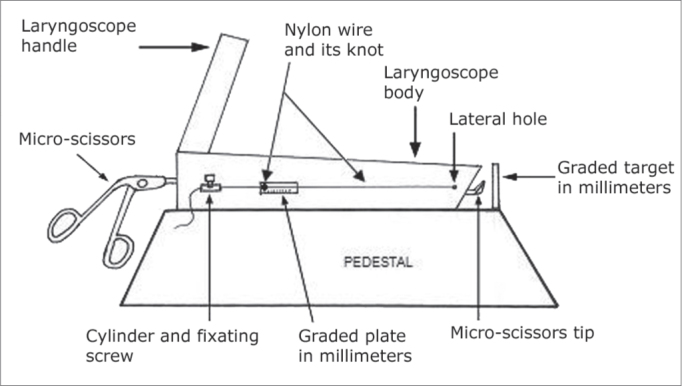
Side view of the laryngoscope and the stabilization system.

**Figure 2:**
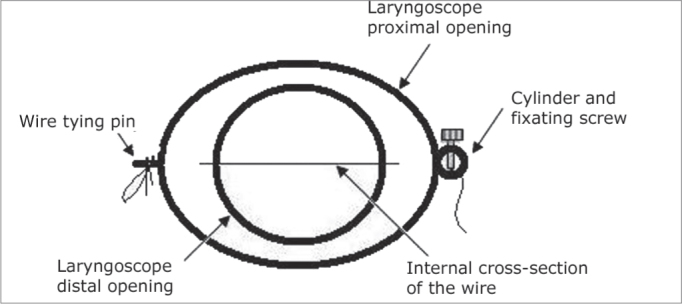
Inside view of the laryngoscope and the horizontal wire.

**Figure 3:**
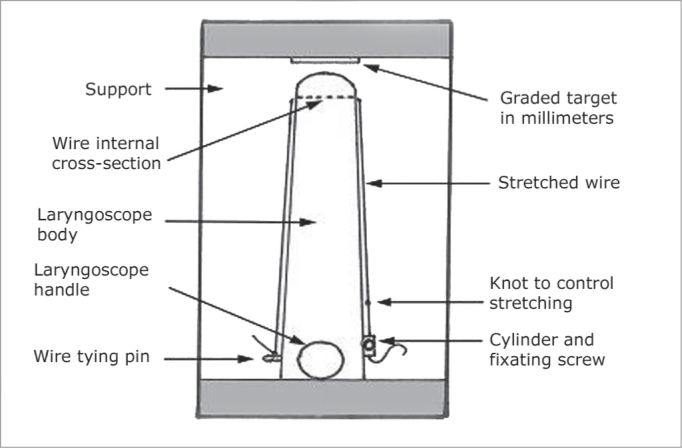
Upper view of the laryngoscope and the stabilization system.

This way, when stretched, the wire is longitudinally positioned on the two lateral faces of the laryngoscope and, inside it, it produces a thin horizontal line cutting in half the middle of the circumference at the distal end, where the microscissors was supported during the tests described below. On the right lateral face the wire would go through the graduated plate. We made a mark (notch) on the wire which, being stretched would coincide with the millimeter zero of the plate.

With the microscissors supported on the horizontal wire, it would suffer a mild lowering because of its elasticity. This lowering was greater in proportion to the laxity of the wire in such a way that, each millimeter of wire laxity would cause an additional lowering. With this lowering, the wire would cause not only vertical stabilization, but also a certain horizontal one. The wire laxity was obtained by loosing the fixation screw and fastening it after repositioning the wire mark on the graduated plate.

The laryngoscope was then fixed to a support and positioned on a table at a convenient height to be used by a surgeon sitting down.

### Study Population

For the tests we used the stabilization system, 10 (ten) physicians with experience in laryngeal microsurgery (at least 10 surgeries done) who volunteered to participate in this study.

None of the participants could have ingested caffeine or done any type of physical exertion in the 24 hours before the test. They also did not have any type of endocrine, neurological, or any other sort of disease which would affect neuromuscular or joint functions.

### Measuring Involuntary Movements

In order to measure the amplitude of the involuntary movements we created a graduated target which was placed at 1 cm (Figs. 1 and 3) in front of the distal end of the laryngoscope. The camera coupled to the microscope would capture the target's image, which was recorded on a DVD for later analysis and measuring of the shifting of the microscissors in relation to the center of the target.

### Test

**Figure 4:**
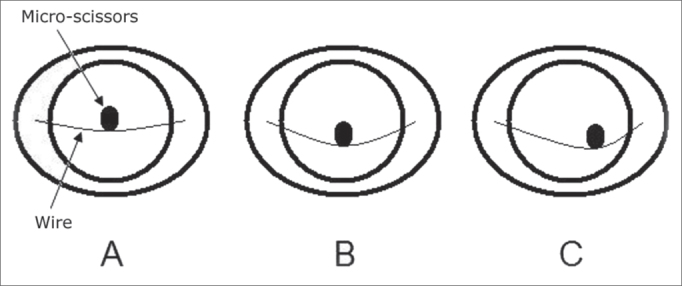
Drawing of the inside of the stabilization system on the 3rd (A), 5th (B) and 6th (C) steps of the test. Notice that even with the wire stretched (A and B), there is a small downwards deflection, which helps on the horizontal stabilization of the microscissors.
4th) with the stabilization system, opening and closing the microscissors, in the middle of the stretched wire;
5th) with the microscissors in the center of the wire and a 3mm loosening, the microscissors still (Fig. 4B).
6th) with the microscissors between the center and the right-side lateral face, on the loose wire at 3 mm, the microscissors was still (Fig. 4C).

Each one of the surgeons was asked to try to keep the microscissors still, with its tip in the center of the graduated target. At each stage of the test, the microscissors's tip should remain between the distal end of the laryngoscope and the target, without touching it for 10 seconds. We asked each one of the surgeons to try and keep the microscissors still, with its tip in the center of the graduated target. At each step of the test, the tip of the microscissors should remain between the distal end of the laryngoscope and the target, without touching it for 10 seconds. One examiner located laterally would make sure the tip of the instrument was properly distant from the target. There should not be any type of elbow support, but the surgeon would support the instrument on the lower border of the distal end of the laryngoscope, simulating a real laryngeal microsurgery situation. The test was carried out in six stages, namely:

1st) without the stabilization system, with the microscissors held still;
2nd) without the stabilization system, opening and closing the microscissors;
3rd) with the stabilization system, with the microscissors still, resting on the center of the stretched wire (mark on zero millimeter) (Fig. 4A);

The target was positioned at each stage of the test so that the center would coincide with the tip of the microscissors at rest. At each stage of the test, as mentioned above, the surgeon should try to keep the instrument centered on the target for 10 (ten) seconds. The target was repositioned at each step of the test so that its center would coincide with the tip of the microscissors at rest. At each step of the test, as aforementioned, the surgeon should have tried to keep the instrument's tip centered on the target during 10 seconds. Therefore, in order to avoid the effect of progressive tiredness, in between each step there was a break of at least 1 minute with the arm at rest.

Using the images recorded on a DVD and analyzing them frame by frame, we wrote down the maximum deviation point of the microscissor's tip in relation to the center of the target during each stage of the test. The images were digitally stored.

### Statistical Analysis

These data (maximum deviations) were statistically analyzed, always comparing the values obtained from the first two steps (without the stabilization system) with the ones found in the other steps. We also compared the results from the fifth and third steps in order to assess the effect of wire loosening in reducing shaking. Finally, we compared the result obtained on the sixth step with that of the fifth in order to estimate the effect the instrument lateralization has on its stabilization. In order to compare the groups we used the t student test for numerical variables with paired samples.

### Ethical Aspects

The study was carried out within scientific ethics standards, and all the participants signed a free and informed consent form, and it was guaranteed to them they would remain anonymous.

## RESULTS

On the first step, without the stabilization system and with the scissors still, the average of maximum deviations found among the 10 surgeons was 2.5 mm. on the second step, without the stabilization system and opening and closing the scissors, this value was 3.5 mm.

On the third step, with the stabilization system and the scissors still at the center of the wire, the mean value of the maximum deviations was 0.8 mm; while for opening and closing the scissors (fourth step), the value was 1.4 mm.

When the microscissors rested still on the center of the loose wire at 3 mm (fifth step), the mean value was 0.4 mm. And when the microscissors was on the right half of the wire (sixth step), the mean value was 0.2 mm.

Tables 1 through 5 showed the comparisons made between the different steps of the test, trying to evaluate the effects of using the system to make up for shaking, as well as the effect of instrument lateralization and the loo- sening of the wire on the stabilization. Chart 1 shows the maximum deviations from each surgeon, on the different steps of the experiment.

**Table 1:** Comparing the mean values between Steps 1 (without the system; still scissors) and 3 (with the system; still scissors in the center; stretched wire), and between Steps 2 (without the system; opening and closing the scissors) and 4 (with the system, opening and closing the scissors; wire stretched).

STEP 1	STEP 3	p	STEP 2	STEP 4	p
2,5 mm	0,8 mm	0,005	3,5 mm	1,4 mm	<0,001

**Table 5:** Comparing the mean values between Steps 3 (with the system; scissors still at the center; stretched wire) and 5 (with the system; scissors at the center; loose wire at 3 mm).

STEP 3	STEP 5	p
0,8 mm	0,4 mm	0,005

**Chart 1:** List of the maximum deviations from each surgeon in each step of the test and the mean values.

SURGEON			STEPS			
	I	II	III	IV	V	VI
1	3,0	4,0	1,0	2,0	0,0	0,0
2	2,0	3,0	0,0	2,0	0,0	0,0
3	2,0	3,0	1,0	2,0	1,0	1,0
4	1,0	2,0	1,0	1,0	1,0	0,0
5	2,0	3,0	1,0	1,0	0,0	0,0
6	4,0	4,0	0,0	0,0	0,0	0,0
7	4,0	5,0	1,0	2,0	1,0	0,0
8	2,0	3,0	0,0	0,0	0,0	0,0
9	3,0	5,0	2,0	3,0	1,0	1,0
10	2,0	3,0	1,0	1,0	0,0	0,0
MEAN	2,5	3,5	0,8	1,4	0,4	0,2

## DISCUSSION

The instrument stabilization system proposed by Armstrong^9^ proved to be very efficient to eliminate shaking during micro-laryngoscopic procedures. Nonetheless, since it is a rigid metal structure, with notches to rest the instrument on, it only allows stabilization in the horizontal direction when the instrument is fit to these notches, reducing shaking, however limiting the surgeon's voluntary movements to one side or the other. On the other hand, since it is not a flexible system, it also limits movements on the anteroposterior direction, thus requiring one more framework placed more inferiorly on the laryngoscope in order to work on the more posterior regions of the glottis; and another one more superiorly in order to work on the anterior glottis.

The system proposed in the present study was also proven efficient in stabilizing the instrument, which can be seen on the results depicted on Table 1, which shows the statistically significant difference between the mean values of deviations obtained in comparing the first and the third steps (p = 0.005) and even more between the second and fourth steps (p < 0.001), showing that, especially during voluntary movements, the stabilization system causes a major reduction in involuntary movements.

In the system hereby proposed, we used a nylon wire, which enables a mild downwards deflection when an instrument rests on it, providing side-to-side stabilization to the instrument, regardless of the instrument's position on the wire. Wherever the instrument rests, due to wire elasticity, there will always be such reflection. This can be well seen on Table 2, which reveals a very significant reduction on the deviation on step 6 when compared to step 1 (p < 0.001), and on Table 3, showing that when the microscissors was positioned on the right side of the wire, there was an average of deviations which was statistically similar to the mean value obtained on the 5th step, with the microscissors in the center and the loose wire at 3 mm (p = 0.2).

**Table 2:** Comparing the mean values between Steps 1 (without the system; still scissors) and 6 (with the system; scissors on the right side; wire loose at 3 mm).

STEP 1	STEP 6	p
2,5 mm	0,2 mm	<0,001

**Table 3:** Comparing the mean values between Steps 5 (with the system; scissors in the center; loose wire at 3 mm) and 6 (with the system; scissors on the right side; loose wire at 3 mm).

STEP 5	STEP 6	p
0,4 mm	0,2 mm	0,2

On the other hand, the system allows not only to reduce shaking, but also the free movement of the instrument along the nylon wire, contrary to what the Armstrong's system does, which, in order to enable side-to-side stabilization it uses notches on the wire, which limits voluntary movements in this direction.

Moreover, the system allows for a fast and easy readaptation to the needs of each disease and for that, all one has to do is to simply vary the wire tensioning. For lesions located on the posterior glottis, we let loose the wire, thus obtaining a deeper arch on the vertex, where the instrument will rest. For lesions located on the anterior glottis, the wire is then tensioned, raising the instrument to a more anterior position. Table 4 shows that there was an important reduction in involuntary movements on step 5 when compared to the first step of the test (p < 0.001), while, on Table 5 we see the loose wire enabling a further stabilization of the instrument than with the tight wire (p = 0.005)).

**Table 4:** Comparing the mean values between Steps 1 (without the system; still scissors) and 5 (with the system; scissors at the center; loose wire at 3 mm).

STEP 1	STEP 5	p
2,5 mm	0,4 mm	<0,001

Shaking is a physiological thing that every surgeon has in a greater or lesser degree. Rarely this shaking can compromise the quality of surgery, especially in microsurgical procedures, which movements must be extremely delicate, as is the case of laryngeal microsurgeries.^1^ In this particular case, no measure can completely eliminate this symptom; nonetheless, some may be taken in order to minimize it.^2^, ^3^, ^4^, ^5^, ^6^, ^7^, ^8^ Among such measures is the development of devices to stabilize the microsurgical instrument. The working principle of such instruments is to reduce the distance between the instrument's fixed point and the site where it will act on the glottis. The longer such distance, broader will be the involuntary movements produced by shaking. Usually in microlaryngoscopy, such fixed point is on the lower border to the proximal opening of the laryngoscope (where the instrument is supported), many centimeters away from the glottis. Both in the Armstrong's system as in ours, not only this fixed point is shifted to a distal site on the laryngoscope, but it also provides for a second support point for the instrument, which can, when convenient, be supported both on the proximal border as on the stabilization system.

It is worth remembering that the system hereby proposed can also be used with other types of wire, such as the cotton or silk wires, which are rougher than nylon, and this can certainly reduce even further the involuntary movements in the side-to-side direction.

By associating the stabilization system with the other measures already known to reduce shaking (arms rest, nutritional care, etc.), we believe it is possible, if not to completely eliminate shaking, but also to satisfactorily reduce it for this type of procedure.

## CONCLUSION

The instrument stabilization system hereby proposed was efficient to reduce shaking in the many situations tested.
